# Outcomes of modern total joint arthroplasty in patients with dementia: a systematic review of challenges and considerations for perioperative care

**DOI:** 10.1007/s00402-026-06233-3

**Published:** 2026-02-24

**Authors:** Halil Bulut, Chuck Lam, Riese Hussain Patel, Tushar Kanti Bhadra, Hassan Tahir, Burcu Bulut-Okay, Erhan Okay, Enes Kanay, Korhan Ozkan

**Affiliations:** 1 Cerrahpaşa School of Medicine, Istanbul University Cerrahpaşa, Istanbul, Turkey; 2 Faculty of Medicine, University of Birmingham, Birmingham, UK; 3 Dhaka Medical College and Hospital, Dhaka, Bangladesh; 4 School of Medicine, Imperial College London, London, UK; 5 Department of Neurology, Fatih Sultan Mehmet Eğitim Ve Araştırma Hastanesi, Istanbul, Turkey; 6 Department of Orthopaedics and Traumatology, Istanbul Goztepe Prof.Dr.Suleyman Yalcin City Hospital, Istanbul, Turkey; 7 Department of Orthopaedics and Traumatology, Acibadem Atasehir Hospital, Istanbul, Turkey

**Keywords:** Dementia, Total joint arthroplasty, Total hip arthroplasty, Total knee arthroplasty, Delirium, Prosthetic joint infection

## Abstract

**Background:**

As the global prevalence of dementia rises, an increasing number of patients with cognitive impairment require Total Joint Arthroplasty (TJA). However, the specific impact of dementia on postoperative outcomes remains underreported. This systematic review evaluates the complications, mortality, and healthcare utilization associated with dementia in patients undergoing Total Hip (THA) and Total Knee Arthroplasty (TKA).

**Methods:**

A systematic search of PubMed, Scopus, Web of Science, Embase, and the Cochrane Library was conducted following PRISMA guidelines. Eligible studies included comparative cohorts of patients with and without a diagnosis of dementia undergoing primary TJA. Methodological quality was appraised using the Newcastle-Ottawa Scale (NOS). Data were synthesized regarding mortality, readmissions, implant-related complications, and discharge disposition.

**Results:**

Seven retrospective cohort studies comprising 13,816 patients with dementia and 869,061 controls were included. The mean NOS score was 8.4/9, indicating high methodological quality. Patients with dementia exhibited significantly worse outcomes across both procedures, including higher rates of postoperative delirium (OR: 4.25–6.40), mortality (HR: 1.43–3.05), and discharge to skilled nursing facilities (OR: 1.87). Stratification by procedure revealed distinct risk profiles: while both cohorts faced high readmission rates, THA patients demonstrated specific vulnerability to mechanical complications, including increased risks of dislocation, periprosthetic fracture (OR: 2.07), and revision surgery. These mechanical failures were frequently driven by falls and poor compliance with postoperative precautions rather than infection alone.

**Conclusion:**

Dementia is a robust independent predictor of adverse outcomes after TJA. While both THA and TKA carry elevated systemic risks, THA poses unique mechanical challenges that may warrant the use of high-stability implants (e.g., dual-mobility cups) to mitigate dislocation risks. Perioperative strategies should prioritize caregiver-led surveillance and strict fall prevention protocols to improve safety in this vulnerable population.

## Introduction

Dementia is a growing global health concern, affecting an estimated 55 million people worldwide, with numbers expected to rise significantly due to aging populations [1]. As life expectancy increases, so too does the likelihood that individuals with cognitive impairment will require orthopedic interventions such as total joint arthroplasty (TJA), including total hip arthroplasty (THA) and total knee arthroplasty (TKA). While TJA remains a highly effective intervention for managing end-stage joint disease, its application in patients with dementia presents unique clinical challenges [2]. Cognitive impairment may influence not only surgical decision-making but also perioperative management, rehabilitation, and long-term outcomes [3].

Patients with dementia are often more frail, present with multiple comorbidities, and may have difficulty adhering to postoperative protocols due to impaired memory and executive function. These factors collectively contribute to a higher risk of complications, increased mortality, prolonged hospital stays, and more frequent discharges to skilled nursing facilities or long-term care settings [4]. Furthermore, perioperative challenges such as delirium, infection, and rehabilitation non-compliance may be exacerbated by cognitive deficits, necessitating tailored care strategies. Despite these concerns, there remains a limited understanding of how dementia specifically affects outcomes following TJA, as the condition is frequently underrepresented or inconsistently reported in large orthopedic datasets [5].

A growing number of observational studies and retrospective analyses have begun to examine the impact of dementia on TJA outcomes. However, findings remain scattered across the literature, with considerable variation in study design, follow-up duration, outcome definitions, and patient populations. Given these inconsistencies and the clinical importance of optimizing care for cognitively impaired patients, a comprehensive synthesis of the existing evidence is warranted.

The primary aim of this systematic review is to evaluate the impact of dementia on outcomes in patients undergoing TJA, including perioperative complications, mortality, functional recovery, hospital length of stay, readmissions, and discharge disposition. Additionally, this review will explore whether perioperative management strategies differ between dementia and non-dementia cohorts. By consolidating current knowledge, this review seeks to inform orthopedic and geriatric practice, identify evidence gaps, and guide future research aimed at improving surgical outcomes for this vulnerable patient population.

## Methods

### Protocol and design

This systematic review was designed to evaluate postoperative outcomes in patients diagnosed with dementia undergoing TJA. The protocol was developed in accordance with the Preferred Reporting Items for Systematic Reviews and Meta-Analyses (PRISMA) 2020 guidelines. A PRISMA flow diagram was used to illustrate the study selection process, and a completed PRISMA checklist was included in the final review [6].

### PROSPERO registration

This systematic review protocol was prospectively registered with PROSPERO (CRD420251163436) and was conducted in accordance with PRISMA guidelines.

### Eligibility criteria

Studies were considered eligible if they involved adult patients with dementia undergoing TJA, specifically THA or TKA. Eligible studies included comparisons either between dementia and non-dementia patients or among subgroups within the dementia population, and reported clinical or surgical outcomes. Acceptable study designs comprised randomized controlled trials, cohort studies, and case-control studies. Only articles published in English and published after 2010 were included. Case reports, reviews, editorials, commentaries, and non-English publications were excluded.

### Information sources

A comprehensive search was conducted in five electronic databases: PubMed, Scopus, Web of Science, Embase, and the Cochrane Library. Additionally, gray literature sources such as dissertations, theses, and relevant conference proceedings were searched. Reference lists of included articles and related reviews were manually screened to identify further relevant studies.

### Search strategy

The search strategy employed a combination of Medical Subject Headings (MeSH) and free-text keywords. For example, the PubMed search included: (“dementia” OR “Alzheimer’s disease”) AND (“total joint arthroplasty” OR “hip replacement” OR “knee replacement”). The Scopus query was: TITLE-ABS-KEY (“total joint arthroplasty” OR “joint replacement”) AND TITLE-ABS-KEY (“dementia” OR “Alzheimer’s disease”) AND TITLE-ABS-KEY (“outcomes” OR “complications” OR “rehabilitation”). The strategy was tailored for each database, and results were imported into reference management software for deduplication.

### Study selection

Two independent reviewers screened all titles and abstracts to assess relevance. Full-text articles were then reviewed for eligibility according to the predefined inclusion criteria. Discrepancies were resolved through discussion or by involving a third reviewer. The selection process was recorded in a PRISMA flow diagram to ensure transparency.

### Data collection process

Data were extracted independently by two reviewers using a standardized data extraction form. Extracted data included study characteristics (author, year, country, study design), patient demographics (age, sex, type and severity of dementia, comorbidities), surgical details (type of joint replacement, surgical approach, perioperative care), and clinical outcomes (complications, mortality, length of stay, discharge destination, and functional recovery). Disagreements in data extraction were resolved by consensus.

### Data synthesis

This review was descriptive in nature. A quantitative meta-analysis was not performed. Instead, findings from eligible studies were synthesized narratively and presented in evidence tables. Differences across study designs, patient characteristics, and reported outcomes were highlighted to contextualize findings.

## Results

### Literature search

**Fig. 1:**
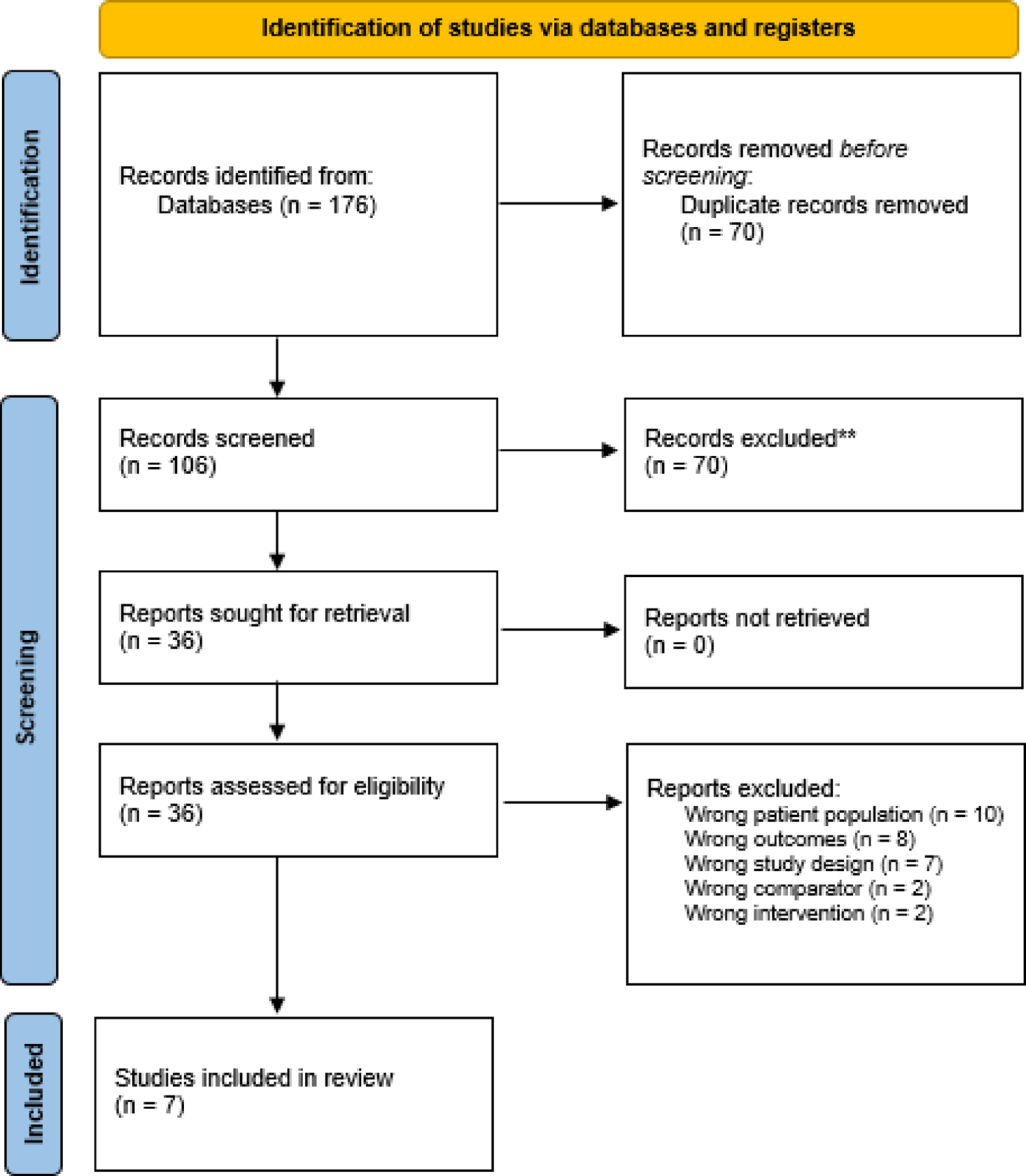
PRISMA flow chart

A comprehensive literature search was conducted across electronic databases, yielding a total of 176 records. Following the removal of 70 duplicates, 106 studies underwent title and abstract screening. Of these, 70 records were excluded due to irrelevance. The remaining 36 full-text articles were retrieved and assessed for eligibility. After detailed evaluation, 29 studies were excluded for reasons including wrong patient population (*n* = 10), outcomes not aligned with the review objectives (*n* = 8), inappropriate study design (*n* = 7), unsuitable comparators (*n* = 2), or interventions not meeting inclusion criteria (*n* = 2). Ultimately, 7 studies fulfilled the eligibility criteria and were included in the final review (Fig. 1).

### Patient cohorts

A total of 13,816 patients with dementia and 869,061 patients without dementia were included across the studies. Among patients with dementia, 7,291 underwent total knee arthroplasty (TKA) and 6,525 underwent total hip arthroplasty (THA). In contrast, among patients without dementia, 299,447 underwent TKA, and 569,614 underwent THA. The Hernandez [8] study presented separate cohorts for TKA and THA, with 1,162 dementia patients undergoing TKA and 247 undergoing THA, while the Johnson [10] study reported an equal distribution of 5,450 patients with and without dementia, with 1558 TKA and 3,892 THA cases in each group. Jämsen [7] included 1,064 dementia patients (619 TKA, 445 THA) and 3,192 non-dementia patients (1857 TKA, 1335 THA). The Rhee [11] study reported 167 dementia patients (84 TKA, 83 THA) compared to 27,199 non-dementia patients (10,039 TKA, 17,160 THA). Finally, Ali [12] presented a large sample with 1858 dementia patients undergoing only THA, and 512,597 non-dementia patients undergoing THA (Table 1).

**Table 1:** Comparison between Non dementia and patients with dementia

Study	Patients with Dementia (*n*)	TKA - Dementia	THA - Dementia	Patients without Dementia (*n*)	TKA - ND	THA - ND
Ottesen [21]	3868	3868	0	152,728	152,728	0
Hernandez **[9]	1162	1162	0	133,265	133,265	0
Jämsen [7]	1064	619	445	3192	1857	1335
Hernandez [9]	247	0	247	34,630	0	34,630
Johnson** [10]	5450*	1558	3892	5450*	1558	3892
Rhee [11]	167	84	83	27,199	10,039	17,160
Ali [12]	1858	0	1858	512,597	0	512,597

*After propensity score matching

** The databases were the same in these studies

### Outcomes following total knee arthroplasty (TKA)

The analysis of Total Knee Arthroplasty outcomes indicates that dementia is associated with significant adverse events, particularly regarding mortality, delirium, and resource utilization. Ottesen [21] demonstrated a correlation between dementia severity and postoperative risks, reporting an increased risk of mortality and delirium that escalates from mild to moderate–severe stages. Additionally, patients with moderate–severe dementia showed an increased risk of requiring intensive interventions (HR: 3.24). In terms of surgical complications and readmissions, Johnson [10] identified an increased risk of 90-day readmission (OR: 1.75) and a trend toward an increased risk of 90-day periprosthetic joint infection (OR: 1.81). Resource utilization was also higher; Hernandez [9] found an increased risk of Emergency Department visits (OR: 1.56) and Skilled Nursing Facility use (OR: 1.87), although this specific study noted that infection and delirium rates were similar to the non-dementia cohort. Finally, Jämsen [7] confirmed a general increased risk of mortality (HR: 1.43) in this population (Table 2).

**Table 2:** Total knee arthroplasty (TKA) outcomes

Study	Key Findings for Knee (TKA)
Ottesen [21]	Mortality and Delirium: Increased risk of mortality in mild dementia (HR: 1.74) Increased risk of mortality in moderate–severe dementia (HR: 3.05) Increased risk of delirium in mild dementia (HR: 4.25) Increased risk of delirium in moderate–severe dementia (HR: 6.40) Increased risk of intensive interventions in moderate–severe dementia (HR: 3.24)
Johnson [10]	Infection and Readmission: Increased risk of 90-day readmission (OR: 1.75) Increased risk of 90-day periprosthetic joint infection trend (OR: 1.81)
Hernandez [9]	Care and ED Visits: Increased risk of Emergency Department (ED) visits (OR: 1.56) Increased risk of Skilled Nursing Facility use (OR: 1.87) *Note: Infection and delirium rates were found to be similar.*
Jämsen [7]	General Mortality: Increased risk of general mortality (HR: 1.43) *(Specific revision risk for TKA was not stated in this study; the mortality finding is common).*

### Outcomes following total hip arthroplasty (THA)

For patients undergoing Total Hip Arthroplasty, dementia was linked to severe complications, including fractures, revisions, and high mortality rates. Johnson [10] highlighted long-term orthopedic risks, reporting an increased risk of proximal femur fracture plating at both 2 years (OR: 2.07) and 5 years (OR: 2.14), alongside an increased risk of 90-day readmission and a trend toward 5-year periprosthetic joint infection. Readmission burdens were further corroborated by Ali [12], who noted an increased risk of all-cause readmission (OR: 1.27) and return-to-theater related readmissions. Regarding implant survival, Jämsen [7] found an increased risk of revision hip arthroplasty specifically in patients with Alzheimer’s Disease (HR: 1.76). Acute postoperative complications were substantial, with Hernandez [9] reporting a marked increased risk of delirium (OR: 5.53) and Emergency Department visits. Furthermore, mortality risks were pronounced in the THA cohort, with Rhee [11] observing a significantly increased risk of mortality (OR: 4.4 vs. 2.6) and Jämsen [7] reporting an increased risk of general mortality (HR: 1.43). (Table 3)

**Table 3:** Total hip arthroplasty (THA) outcomes in patients with dementia

Study	Key Findings for Hip (THA)
Johnson [10]	Fracture, Infection, and Readmission: Increased risk of 90-day readmission (OR: 2.17) Increased risk of 2-year proximal femur fracture plating (OR: 2.07) Increased risk of 5-year proximal femur fracture plating (OR: 2.14) Increased risk of 5-year periprosthetic joint infection trend (OR: 1.48)
Ali [12]	Readmission: Increased risk of all-cause readmission (OR: 1.27) Increased risk of return to theater (RTT)-related readmission (OR: 1.57)
Jämsen [7]	Revision and Mortality: Increased risk of revision hip arthroplasty in Alzheimer’s Disease (HR: 1.76) Increased risk of general mortality (HR: 1.43)
Hernandez [9]	Delirium and ED Visits: Increased risk of delirium (OR: 5.53) Increased risk of Emergency Department (ED) visits (OR: 1.96)
Rhee [11]	Mortality: Increased risk of mortality after THA (OR: 4.4 vs. 2.6)

### Quality assessment

The methodological quality of the included studies was assessed using the Newcastle-Ottawa Scale (NOS), yielding a mean score of 8.4 out of 9, indicating a high overall quality of evidence. Four studies—Ottesen et al. [21], Johnson et al. [10], Rhee et al. [11], and Jämsen et al. [7]—achieved a maximum score of 9/9, demonstrating exceptional rigor in selection, comparability, and outcome assessment. Hernandez et al. [9] received a high-quality rating of 8/9 for both their TKA and THA analyses, while Ali et al. [12] were classified as good quality with a score of 7/9. Notably, every study received the maximum score (4/4) in the “Selection” domain, reflecting the high representativeness of the large-scale administrative databases and registries utilized. Variations in scoring were primarily driven by the “Comparability” domain, where studies utilizing robust propensity score matching (e.g., Johnson, Jämsen) received higher ratings compared to those relying solely on multivariate regression (Table 4).

**Table 4:** Newcastle-Ottawa scale (NOS) quality assessment

Study	Selection (Max 4)	Comparability (Max 2)	Outcome (Max 3)	Total Score
Ottesen [21]	4	2	3	9 / 9
Johnson [10]	4	2	3	9 / 9
Hernandez [9] *(TKA)*	4	1	3	8 / 9
Hernandez [9] *(THA)*	4	1	3	8 / 9
Rhee [11]	4	2	3	9 / 9
Ali [12]	4	1	2	7 / 9
Jämsen [7]	4	2	3	9 / 9

**Table 5:** Evidence-Based recommendations for perioperative management of TJA in patients with dementia

Phase	Focus Area	Clinical Recommendations & Actions
Pre-operative	Assessment & Selection	Cognitive Screening: Use *Mini-Cog* or *AMTS*. Frailty: Use *Edmonton Frail Scale* to stratify delirium risk. Approach: Dementia is not a contraindication, but requires a “selected patient” approach.
	Informed Consent	Risk Discussion: Use data (e.g., *Rhee et al.*) to clarify that while pain relief is achievable, mortality risks are significantly higher. Family Expectation: Establish realistic goals regarding discharge destinations (SNF vs. Home).
	Medication Management	Continue: Donepezil. Withhold: Rivastigmine/Galantamine (1 day prior) if neuromuscular blockade is anticipated. Avoid: Benzodiazepines, tramadol, and anticholinergics due to deliriogenic potential.
	Prehabilitation	Engagement: Involve family in physical/cognitive prehab. Communication: Utilize the “This is me” form to aid staff in personalized communication and orientation.
Intra-operative	Anesthesia Strategy	Agents: Propofol TIVA is preferred over volatile agents (sevoflurane) to reduce amyloid aggregation risk. Monitoring: Use processed EEG and age-adjusted MAC titration to prevent anesthetic overdose.
	Surgical Technique	Implant Choice: Given the high risk of dislocation (*Jämsen et al.*), prioritize high-stability implants (e.g., dual-mobility cups, larger femoral heads), particularly for THA.
	Environment	Optimization: Schedule cases early in the day in a quiet, well-lit room. Support: Allow caregiver presence during induction to reduce anxiety.
Post-operative	Delirium & Pain	Screening: Daily delirium screening (e.g., 4AT) for the first 72 h. Pain Assessment: Use observational tools like the Abbey Pain Scale for non-verbal patients. Analgesia: Prioritize opioid-sparing, multimodal analgesia.
	Discharge Planning	Placement: Initiate Skilled Nursing Facility (SNF) discussions immediately upon admission (OR for SNF: 1.87). Paradigm Shift: Do not rely on “patient compliance.” Shift to caregiver-led surveillance for wound care.
	Safety Protocols	Fall Prevention: Implement strict fall prevention protocols rather than aggressive independent mobilization to mitigate the doubled risk of periprosthetic fractures (*Johnson et al.*).

## Discussion

Analysis of seven large-scale studies involving 13,816 patients with dementia and 869,061 without revealed consistently poorer outcomes for dementia patients undergoing total joint arthroplasty (TJA). Across both total knee (TKA) and total hip arthroplasty (THA), dementia was associated with higher complication rates, mortality, and healthcare utilization. Hernandez et al. [9] reported increased emergency department visits (OR: 1.56–1.96), greater skilled nursing facility use (OR: 1.87), and significantly elevated delirium risk (OR: 5.53). Jämsen et al. [7] found a higher revision risk (HR: 1.76) and increased mortality (HR: 1.43) in patients with Alzheimer’s disease. Johnson et al. [10] demonstrated elevated 90-day readmissions after both TKA (OR: 1.75) and THA (OR: 2.17), with trends toward higher periprosthetic joint infections and long-term femur fracture risk. Rhee et al. [11] observed greater mortality post-THA (OR: 4.4 vs. 2.6) and longer hospital stays in dementia patients. Ali et al. [12] identified increased all-cause (OR: 1.27) and treatment-related readmissions (OR: 1.57). These findings underscore dementia as a major risk factor for worse clinical outcomes, emphasizing the need for specialized perioperative management, early discharge planning, and coordinated post-acute care in this vulnerable population.

### Pathophysiological and functional mechanisms

The heightened risk of complications in dementia patients undergoing TJA is underpinned by a complex interaction between biological vulnerabilities and functional deficits. Biologically, the “fragile brain” in dementia is characterized by chronic microvascular dysfunction, including endothelial injury and increased blood–brain barrier permeability [13]. These alterations render the brain highly susceptible to perioperative insults common in orthopedic surgery, such as hemodynamic fluctuations, fat emboli released during marrow instrumentation [14–16], and neuroinflammatory responses triggered by anesthesia and surgical stress [17–19]. Together, these mechanisms explain the disproportionately high rates of postoperative delirium observed in this population.

However, while biological factors drive acute cognitive dysfunction, functional and behavioral mechanisms appear to be the primary drivers of the elevated revision rates observed in this review. Cognitive decline significantly compromises the patient’s ability to adhere to postoperative restrictions. Jämsen et al. [7] identify this inability to follow weight-bearing or range-of-motion precautions as a leading cause of the increased risk of dislocation and subsequent revision in THA. Furthermore, the inherent neuromuscular deficits and gait instability in dementia patients exacerbate fall risk; Johnson et al. [10] attribute the two-fold increase in revision surgery not to infection, but specifically to periprosthetic fractures resulting from postoperative falls. Finally, as noted by Rhee et al. [11], dementia serves as a marker for generalized frailty and depleted physiological reserve, rendering these patients less capable of recovering from surgical stress, thereby contributing to higher mortality rates.

### Prevalence and clinical importance of dementia

The prevalence of dementia rises sharply with age: approximately 5–7% in individuals over 65, 15% in those over 75, and up to 30% in those over 85 [20]. Given that TJA is most commonly performed in this same demographic, dementia represents an increasingly common and clinically significant factor in arthroplasty practice. Its growing prevalence magnifies not only surgical risks but also the burden on healthcare systems, complicating discharge planning and long-term care requirements.

### Impact of dementia on postoperative complications

Patients with dementia are at an increased risk of specific postoperative complications, notably delirium and also potentially prosthetic joint infection (PJI). According to Hernandez et al. [9], the risk of delirium is particularly pronounced, with odds ratios as high as 5.53. It is likely a result of the population’s low neurocognitive reserve and is associated with poor functional recovery, longer hospitalization, and increased mortality. The data on PJI is less consistent, though Johnson et al. [10] noted a trend toward increased infection rates, which may be attributable to compromised hygiene, wound care, and immune function. These findings emphasize the need for early delirium screening, infection prevention protocols, and caregiver education to mitigate avoidable harm in cognitively impaired surgical patients.

### Mortality and long-term outcomes

Dementia is associated with increased short- and long-term mortality following TJA, with the reported risk ranging from a 43% increase (HR: 1.43) in patients with Alzheimer’s disease Jämsen et al. [7] to a more than four-fold increase in post-THA mortality Rhee et al, [11]. This excess mortality may reflect the cumulative effects of frailty, limited physiological reserve, and poor recovery potential, compounded by perioperative complications. Long-term risks, including periprosthetic fractures Johnson et al., [10] and revision surgery Jämsen et al. [7], also increase with cognitive decline, undermining the durability of TJA. The interaction between dementia and comorbidities likely amplifies these risks. These findings support a more conservative and carefully individualised approach to TJA in dementia patients, especially those with advanced disease.

### Hospital length of stay and discharge planning

Dementia patients typically have prolonged hospital stays Rhee et al. [11] and are more often discharged to skilled nursing facilities rather than returning home. These extended stays are often due to mobilisation delays, increased postoperative confusion, and the need for social and medical stabilization. The high rates of discharge to institutional care, with odds ratios of 1.87 Hernandez et al. [9], highlight the challenges in arranging safe home discharge for patients with impaired cognition. Continuity of care becomes fragmented, increasing the risk of readmission and delayed rehabilitation. Proactive discharge planning involving social workers, geriatricians, and physiotherapists is essential to ensure safe and effective transitions of care.

### Challenges in perioperative management and rehabilitation

Cognitive impairment impedes understanding of postoperative instructions, adherence to physiotherapy, and engagement in rehabilitation. These are all critical to successful recovery after TJA. Patients with dementia often struggle with pain communication, experience sensory overload in hospital settings, and are at higher risk of postoperative delirium, according to findings from Hernandez et al. [9]. These barriers require customized care protocols, including routine cognitive screening, enhanced pain management, and early mobilization strategies. Non-pharmacological delirium prevention, use of patient navigators, and caregiver involvement should be integrated into perioperative planning. Without targeted interventions, these patients face a cyclical pattern of complications, delayed recovery, and functional decline.

### Variability in study designs and reporting

The heterogeneity of the included studies limits generalisability and complicates this review. Studies varied widely in population demographics, dementia severity, surgical approach, outcome definitions, and follow-up duration. Some relied on administrative codes without clinical validation of dementia diagnoses or surgical outcomes, risking a potential misclassification bias. Inconsistent reporting of functional outcomes and rehabilitation adherence further obscures the real-world impact. These limitations highlight the urgent need for standardised reporting frameworks, stratification by dementia severity, and inclusion of patient-centered outcomes. Until then, clinicians must interpret existing data cautiously, using it as a tool to inform, but not solely dictate, individualised care decisions.

### Clinical implications and recommendations for practice

#### Pre-operative phase

Dementia should not be considered a contraindication for total joint arthroplasty (TJA); patients with cognitive impairment deserve equal access and perioperative standards of care. Preoperative evaluation must include brief cognitive screening (Mini-Cog or AMTS) and frailty assessment (Edmonton Frail Scale) to stratify postoperative delirium and cognitive decline risk. Cholinesterase inhibitors should be managed carefully — donepezil is typically continued, whereas rivastigmine or galantamine may be withheld one day before surgery if neuromuscular blockade is anticipated. Benzodiazepines, tramadol, and anticholinergics should be avoided due to their deliriogenic potential. Incorporating family-involved prehabilitation, including physical activity, cognitive stimulation, and nutritional optimization, significantly lowers delirium rates (19.4% → 2.6%). Active caregiver participation and personalized communication strategies, such as the “This is me” form, improve perioperative continuity and orientation [22, 23] (Table 5).

#### Intra-operative phase

There is no significant difference in cognitive outcomes between general and regional anesthesia, but the emphasis should be on minimizing deliriant exposure and maintaining physiological stability. Propofol is preferred over volatile anesthetic agents (e.g., sevoflurane, isoflurane) due to its lower association with amyloid aggregation. Processed EEG monitoring and age-adjusted MAC titration should be used to avoid anesthetic overdose, especially in cognitively vulnerable patients. Maintaining hemodynamic stability, normoxia, normothermia, and correcting metabolic imbalances are crucial. Opioid-sparing multimodal analgesia, complemented by regional nerve blocks such as femoral or adductor canal techniques, helps reduce postoperative confusion. A dementia-friendly operating room—quiet, well-lit, scheduled early in the day, and allowing caregiver presence—can mitigate anxiety and disorientation [22, 23]. Given the heightened risk of dislocation due to poor compliance, surgeons should consider high-stability implant options, such as dual-mobility cups or larger femoral heads, particularly in THA. Additionally, the informed consent process must be robust; using data from Rhee et al. and Jämsen et al., clinicians should engage in transparent risk-benefit discussions with families, clarifying that while dementia is not an absolute contraindication, it necessitates a ‘selected patient’ approach to weigh pain relief against significantly higher mortality risks.

#### Post-operative phase

Postoperatively, patients should undergo daily delirium screening (e.g., 4AT) during the first 72 h, as early detection enables prompt management. Cholinesterase inhibitors and memantine should be restarted once clinically stable. Non-opioid multimodal analgesia should be prioritized, and pain in non-verbal patients should be assessed using tools such as the Abbey Pain Scale. Early mobilization, adequate hydration, and regular bowel and bladder care facilitate recovery (21,22). However, discharge planning requires a paradigm shift based on the findings of this review. Hernandez et al. [9] demonstrate that discharge to skilled nursing facilities (SNF) is frequently required (OR: 1.87); thus, realistic discussions with families regarding placement must occur immediately. Crucially, to mitigate the long-term risks identified by Johnson et al. [10]—specifically periprosthetic fractures and infections—the post-discharge regimen should prioritize strict fall prevention protocols and caregiver-led wound surveillance, rather than relying on patient compliance.

### Future research directions

This review highlights a clear lack of prospective, high-quality research evaluating perioperative interventions specific to dementia patients undergoing TJA. Future studies should stratify outcomes based on dementia type and severity, assess functional and quality-of-life metrics, and evaluate the effectiveness of tailored care models. The role of caregiver involvement, rehabilitation compliance, and novel delirium prevention strategies also needs more study. Standardised definitions of complications and longer follow-up periods are essential to capture the full impact of TJA in this vulnerable group.

### Limitations of this review

This study has several limitations. The inclusion of varied study designs—predominantly retrospective and observational cohorts—introduces significant heterogeneity and a high susceptibility to confounding bias, which may limit the generalizability of the findings. Due to this heterogeneity, a quantitative meta-analysis was not performed, which prevented the calculation of pooled effect sizes or a formal analysis of publication bias. Secondly, all included studies were observational and retrospective, making them susceptible to confounding variables and selection bias that may not have been fully addressed. Thirdly, our search was restricted to English-language publications, potentially introducing language bias and omitting relevant studies from other regions. Finally, variation in follow-up periods across the studies may affect the reliability of the assessment of long-term outcomes. We also note the potential for publication bias, where studies with statistically insignificant findings are less likely to be published, and this cannot be entirely ruled out.

## Conclusion

In conclusion, this systematic review finds that a diagnosis of dementia is a significant predictor of adverse outcomes following total joint arthroplasty, due to a myriad of factors such as a substantially higher risk of postoperative complications (such as delirium) facing this patient group. Increased long-term mortality and implant failure rates are also noted. Our findings point to a need for tailored multi-disciplinary perioperative care pathways for cohesion across orthopaedic surgeons, geriatricians, and community care: proactive risk stratification is essential for preventing complications arising postoperatively for this vulnerable patient group.

## Data Availability

No datasets were generated or analysed during the current study.
